# Regionally Distinct Alterations in the Composition of the Gut Microbiota in Rats with Streptozotocin-Induced Diabetes

**DOI:** 10.1371/journal.pone.0110440

**Published:** 2014-12-03

**Authors:** Roland Wirth, Nikolett Bódi, Gergely Maróti, Mária Bagyánszki, Petra Talapka, Éva Fekete, Zoltán Bagi, Kornél L. Kovács

**Affiliations:** 1 Department of Biotechnology, University of Szeged, Szeged, Hungary; 2 Department of Physiology, Anatomy and Neuroscience, University of Szeged, Szeged, Hungary; 3 Institute of Biochemistry, Biological Research Centre, Hungarian Academy of Sciences, Szeged, Hungary; 4 Institute of Biophysics, Biological Research Centre, Hungarian Academy of Sciences, Szeged, Hungary; 5 Department of Oral Biology and Experimental Dentistry, University of Szeged, Szeged, Hungary; Charité, Campus Benjamin Franklin, Germany

## Abstract

The aim of this study was to map the microbiota distribution along the gut and establish whether colon/faecal samples from diabetic rats adequately reflect the diabetic alterations in the microbiome. Streptozotocin-treated rats were used to model type 1 diabetes mellitus (T1D). Segments of the duodenum, ileum and colon were dissected, and the microbiome of the lumen material was analysed by using next-generation DNA sequencing, from phylum to genus level. The intestinal luminal contents were compared between diabetic, insulin-treated diabetic and healthy control rats. No significant differences in bacterial composition were found in the luminal contents from the duodenum of the experimental animal groups, whereas distinct patterns were seen in the ileum and colon, depending on the history of the luminal samples. Ileal samples from diabetic rats exhibited particularly striking alterations, while the richness and diversity obscured some of the modifications in the colon. Characteristic rearrangements in microbiome composition and diversity were detected after insulin treatment, though the normal gut flora was not restored. The *Proteobacteria* displayed more pronounced shifts than those of the predominant phyla (*Firmicutes* and *Bacteroidetes*) in the rat model of T1D. Diabetes and insulin replacement affect the composition of the gut microbiota in different, gut region-specific manners. The luminal samples from the ileum appear more suitable for diagnostic purposes than the colon/faeces. The *Proteobacteria* should be at the focus of diagnosis and potential therapy. *Klebsiella* are recommended as biomarkers of T1D.

## Introduction

Type 1 diabetes (T1D) is an autoimmune disease that results from the T cell-mediated destruction of insulin-producing beta cells [1]. Recent studies suggest that there might be an inflammation-triggering effect of the intestinal microbiota in the development of autoimmune diabetes [2]–[5]. The link between the gut microbiota and the development of autoimmune diabetes can be explained by the shared lymphocyte-homing receptors in the gut and inflamed pancreas [6]. T1D is frequently associated with a variety of gastrointestinal (GI) motility abnormalities in which selective nitrergic myenteric neuropathy has been well documented both in humans [7]–[9] and in rodent models [10]–[12]. Nevertheless, diabetes-related enteric neuropathy as an immune-mediated disease has received less attention. We earlier demonstrated [10]–[11] that myenteric neurones and microvessels adjacent to the myenteric ganglia are direct targets of diabetes, and the rate and extent of their damage depend strictly on the intestinal segment in which the particular neurones or capillaries are located. Moreover, their responsiveness to insulin replacement is also gut region-dependent. These observations indicated a diabetes-related pathological microenvironment, allowing neurones to survive in a strictly intestinal segment-specific way, even under appropriate glycaemic control. Several studies have indicated that, in addition to being targets of inflammation, the peptidergic enteric neurones modulate the immune cell function and can therefore stimulate pro-inflammatory cytokine production and result in neurodegeneration [13]–[15]. These data suggest that the pathogenic cascade which leads to the development of autoimmune diabetes through secreted lymphokines might also result in altered neuro-immune interactions and provoke myenteric neuropathy. Of the potential environmental triggers implicated in the development of diabetes-related myenteric neuropathy, the intestinal microbiome is regarded as primary candidate.

The microbiome of the gut has been extensively studied, particularly in humans, during the past few years, including major metagenomic projects in the USA [16]–[19] and Europe [20]–[22]. A major limitation of these mega efforts is the fact that the overwhelming majority of the samples used to study microbial events in the gut were taken from stools, i.e. material from the very end of food processing. The underlying assumption that all microbes thriving in the gut are equally represented in the stools is at best only partially correct [23]–[27]. The relative simplicity and non-invasive nature of this sampling method remain the main justifications of this approach.

In consequence of the difficulties in sample collection, much less is known about the composition of the microbiota in the duodenum, jejunum and ileum in healthy or various disease states. Although the available data indicate a gut region-specific composition of the microbiota associated with health and GI disorders, this situation is frequently disregarded [23]–[28].

Our study was inspired by the findings that myenteric neurones and microvessels in different intestinal segments display various susceptibilities to diabetic damage and also exhibit different responses to insulin treatment. In an attempt to search for a causal relationship between the prevalence of bacteria in the specific parts of the GI tract and the region-specific pathological microenvironments, we investigated the spatial distribution of the microbes along the gut of diabetic and insulin-treated diabetic rats relative to healthy controls.

## Materials and Methods

### Animal model

Adult male Wistar rats (Crl:WI BR; Toxi-Coop Zrt.) weighing 290–300 g, kept on standard laboratory chow (BioplanKft.) and with free access to drinking water, were used throughout the experiments. The rats were divided randomly into three groups: animals with STZ-induced diabetes (n = 8), animals with insulin-treated diabetes (n = 8), and sex- and age-matched controls (n = 6). Hyperglycaemia was induced by a single intraperitoneal injection of STZ (Sigma, USA) [10]. Forty-eight hours later, the nonfasting blood glucose concentration was determined in blood obtained from the cut tip of the tail by the glucose oxidase method, using a portable blood glucose monitoring device (D-Cont Personal, 77 Elektronika Kft, Hungary). The animals were considered diabetic if the non-fasting blood glucose concentration was>18 mM [10]–[11]. From this time on one group of hyperglycaemic rats received a subcutaneous injection of insulin (Humulin M3; Eli Lilly Nederland) each morning (4 U) and afternoon (2 U). The non-fasting blood glucose concentration and weight of each animal were measured weekly. In all procedures involving experimental animals, the principles of laboratory animal care (NIH Publication No. 85-23, revised 1985) were strictly followed, and all the experiments were approved in advance by the Local Ethics Committee for Animal Research Studies at the University of Szeged.

### Tissue handling and collection of intestinal contents

Ten weeks after the onset of diabetes, the animals were killed by cervical dislocation under chloral hydrate anaesthesia (375 mg/kg i.p). The gut segments of the rats in the control, STZ-induced diabetes and insulin-treated diabetes groups were dissected and rinsed in sterile distilled water (Milli-Q). Samples were taken from the duodenum (10-cm-long samples from distal to the pylorus), the ileum (10-cm-long samples from proximal to the ileo-caecal junction), and the entire colon and processed for metagenomic studies. For the collection of intestinal contents, the dissected gut segments were washed thoroughly twice with a strong jet of sterile distilled water (2×10 ml, Milli-Q). This combined solution was shaken in sterile Falcon tubes and divided into 2 ml aliquots, which were and frozen at −80°C until DNA extraction.

### DNA isolation for metagenomic studies

The lumen contents of each gut segments (3×2 ml) were used to prepare the total community DNA. The cells were lysed in three different lysis solutions (Table 1); enzymatic (lysozyme), chemical (cetyltrimethylammonium bromide, CTAB) and physical (heat or mechanical) cell wall disintegration methods were employed. The lysis conditions were optimized for each GI segment. Samples from the duodenum and ileum were incubated at 37°C for 1 h to complete cell disruption in each lysis solution (A, B and C in Table 1); the more concentrated colon samples were treated with bead disintegration. After lysis, samples from the duodenum and ileum were mixed with 125 µl (duodenum) or 200 µl (ileum) Qiagen QIAamp Stool AL (Qiagen, 51504) buffer and 25 µl proteinase K (Panreac Applichem GmbH, A3830), and were further incubated at 56°C for 1 h. The DNA samples were next centrifuged (12,000 rpm, 2 min) and the DNA from the supernatant was precipitated with 400 µl chilled ethanol. In the following steps, the manufacturers’ instructions were followed as given in the Qiagen QIAamp DNA Stool DNA (duodenum and ileum) and Zymo Research Fecal DNA miniprep (Zymo Research Europe) (colon) kits, respectively. Finally, the DNA isolated from the various GI segments with each lysis method was combined. The DNA preparations from the intestinal segments of 3 rats for each condition were isolated separately and parallel samples were pooled for sequencing.

**Table 1:** Cell lysis conditions applied to obtain maximum DNA for the next-generation sequencing of the microbial community in the various segments of the GI tract.

GI tractsegment		Lysozyme^1^[µl]	10% CTAB^2^[µl]	genomic CTABlysis buffer^3^ [µl]	Qiagenbuffer^4^ [µl]	Zymobuffer^5^ [µl]	Heat/Beaddisruption
Duodenum	A	–	50	–	–	150	Heat
	B	50	–	–	150	–	Heat
	C	50	–	150	–	–	Heat
Ileum	A	-	50	–	–	250	Heat
	B	50	–	–	250	–	Heat
	C	50	–	250	–	–	Heat
Colon	A	-	100	–	100	550	Bead
	B	250	100	–	100	300	Bead
	C	250	–	300	200	–	Bead

1 100 mg/ml (Applycehm). ^2^Cetyltrimethylammonium bromide (w/v). ^3^1 M Tris-HCl 100 ml, 500 mM EDTA 50 ml, 5 M NaCl 300 ml, 10% CTAB, 20% SDS, pH = 8^29^. ^4^ASL buffer from Qiagen QIAamp DNA Stool miniprep kit (Qiagen, 51504). ^5^From Zymo Research Fecal DNA kit (Zymo Research, D6010).

The quantity of DNA was determined in a NanoDrop ND-1000 spectrophotometer (NanoDrop Technologies) and Qubit 2.0 Fluorometer (Life Technologies). DNA purity was tested by agarose gel electrophoresis and by Agilent 2200 Tape Station (Agilent Technologies).

### Next-generation DNA sequencing and data handling

The sample preparation for total metagenome sequencing of the pooled samples was carried out following the recommendations of Ion Torrent PGM sequencing platform (Life Technologies). Sequencing was performed using Ion Torrent PGM 316 chips [29]. The reads were analysed and quality values were determined for each nucleotide. The 100–200 nucleotide long individual sequences were further analysed by using the MG-RAST software package, which is a modified version of RAST (Rapid Annotations based on Subsystem Technology). The MG-RAST server initially runs a quality control test. If the data appear reliable, the system automatically screens for sequences of potential protein encoding regions via a BLASTX search against the comprehensive non-redundant database compiled from various publicly available sequencing centres and other sources. These databases include several rDNA datasets, e.g. GREENGENES, RDP II and European 16S RNA, among other information sources. The generated matches to external databases were used to compute the derived data. Details of the statistical calculations were published [29].

## Results

### Weight and glycaemic characteristics of experimental animals

Ten weeks after the onset of diabetes, the diabetic rats were characterized by a reduced body weight and an increased blood glucose concentration as compared with the age- and sex-matched controls. The insulin-treated diabetic rats did not differ significantly from the control animals in weight or blood glucose concentration (Table 2).

**Table 2:** Weight and glycaemic characteristics of the three experimental groups of rats.

Rat group	Body weight (g) ± SEM		Blood glucose concentration (mM) ± SEM	
	initial	final	initial	final
Controls (n = 6)	292.7±1.8	417.0±13.9***	6.3±0.2	5.4±0.1
Diabetics (n = 8)	297.8±5.8	347.5±19.3*	5.9±0.3	30.8±1.6***
Insulin-treated diabetics (n = 8)	299.3±5.2	397.3±9.6 ***	5.9±0.3	4.0±0.6

Initial vs. final: *p<0.05; ***p<0.001.

### The composition of the microbiome along the gut

**Figure 1:**
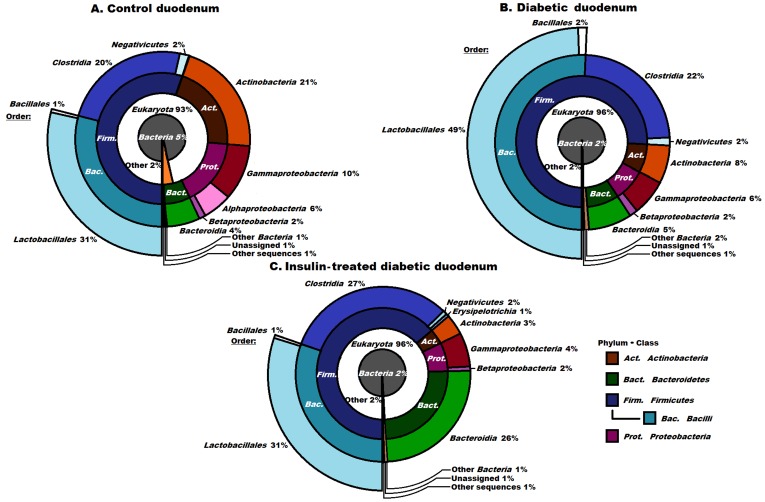
Compositions of the control, diabetic and insulin-treated diabetic bacterial microbiomes in the duodenum at domain, phylum and class levels. Only the predominant order *Lactobacillales* is indicated. The abbreviated and colour-coded taxa are indicated on the right side in systematic sequence. “Other sequences” are probably those of viruses not covered in the databases used. The majority of the identified DNA sequences related to the *Eukaryota*, indicating the overall low abundance of microbes. Among the *Bacteria* the *Lactobacillales* predominated. No significant differences were observed between the control and the diabetic and insulin-treated diabetic samples.

**Figure 2:**
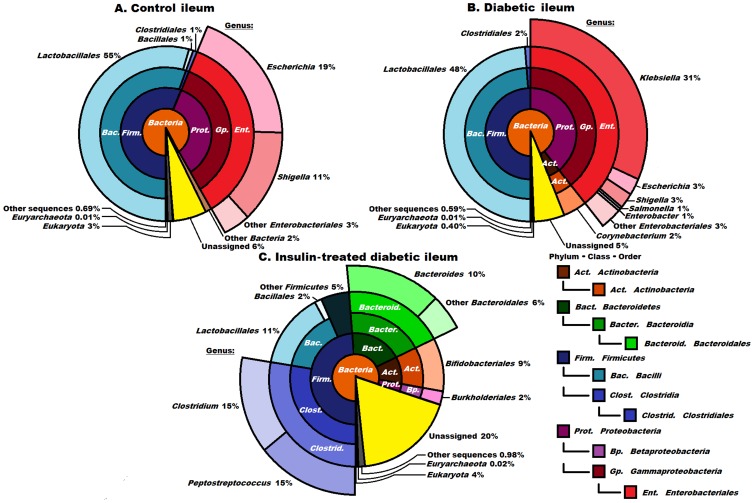
Compositions of the control, diabetic and insulin-treated diabetic microbiomes in the ileum at domain, phylum, class and order levels. The orders *Enterobacteriales* (2B), *Clostridiales* and *Bacteroidetes* (2C), displaying the most striking differences, are shown in higher resolution. The abbreviated and colour-coded taxa are indicated on the right side in systematic sequence. “Other sequences” are probably those of viruses not covered in the databases used. Taxa with very low (<1%) abundances are indicated to two decimal places. A striking invasion of the genus *Klebsiella* was apparent in diabetes. It is noteworthy that the insulin treatment eliminated the *Klebsiella* invasion, but did not restore the “healthy” microbiome, and the representation of the classes *Clostridia* and *Bacteroidia* increased massively.

**Figure 3:**
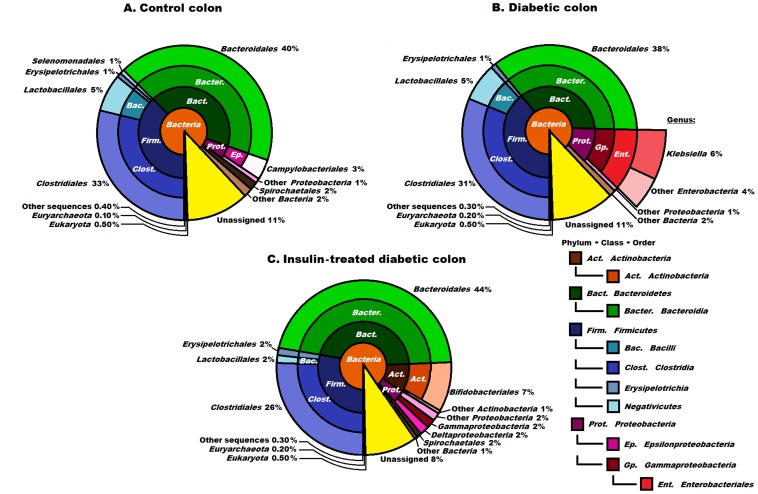
Compositions of the control, diabetic and insulin- treated diabetic microbiomes in the colon at domain, phylum, class and order levels. The abbreviated and colour-coded taxa are indicated on the right side in systematic sequence. Two minor classes belonging in the phylum *Firmicutes* are marked only with colours. “Other sequences” are probably those of viruses not covered in the databases used. Taxa with very low (<1%) abundances are indicated to two decimal places. The diversity of the microbial community was increased significantly in the insulin-treated diabetic animals, members of the order *Bacteroidales* predominating. The order *Enterobacteriales* is shown at higher resolution in diabetes (3B). The presence of the *Klebsiella* in the diabetic samples was still clearly seen although not as markedly as in the case of the ileum. Insulin promoted the abundance of the order *Bifidobacteriales*.

Two sets of metagenomic data were evaluated throughout the present study. The distributions at phylum, class and order levels (Figures 1–3) included all DNA reads comprising the eukaryotic sequences and unassigned ones. The latter indicated the number of sequences that could not be assigned to any known prokaryotic genome uploaded onto publicly available databases. In this way, we intended to present both the variations due to unrelated or unidentifiable data and the distribution of abundances among the bacterial taxa. The most important results are indicated at a genus level for the control, the diabetic and insulin-treated ileum in figure 2 and for diabetic colon in Figure 3.

### The microbiome of the duodenum

In line with the low abundance of the microbiological community in this region, the overwhelming majority of the DNA sequences from the duodenal samples related to rodents (93–96%, Figure 1). The few prokaryotes comprised mostly members of the phylum *Firmicutes* and class *Bacilli*. When the eukaryotic and unidentified sequences were removed from the database, 31–49% of the total reads were due to the order *Lactobacillales*, comprising almost entirely members of the genus *Lactobacillus* (data not shown). The richness and distribution of prokaryotes did not differ markedly in the diabetic and insulin-treated diabetic rats relative to the healthy controls (Figure 1).

### The microbiome of the ileum

#### The microbiome of the control ileum

Only 3% of the DNA sequences originated from eukaryotic cells in this segment. The ileum was populated by *Bacteria*, but representatives of *Archaea* (0.01%) also occurred (Figure 2A). The majority of the domain *Bacteria* belonged in the phyla *Firmicutes* (57%) and *Proteobacteria* (about 30%). The majority of the *Firmicutes* were identified in the class Bacilli, order *Lactobacillales* (55%) and genus *Lactobacillus* (54%). The genera *Escherichia* (19%) and *Shigella* (11%) predominated in the *Proteobacteria*.

#### The microbiome of the diabetic ileum

Striking differences were observed in the composition of the microbial community of the ileal lumen between the diabetic rats and the controls (Figures 2A and 2B). Among the *Firmicutes* (50%), the genus *Lactobacillus* predominated (48%) and *Streptococcus* (2%) was also detected, the distribution strongly resembling that in the control ileum. The *Actinobacteria* (not observed in the controls) appeared with representatives of the order *Corynebacterium* (2%). The total ratio of *Proteobacteria* was not changed greatly (42% as compared with 33% in the control ileum), but a massive invasion by the genus *Klebsiella* (31%) led to its predominance in the bacterial sequences in the diabetic ileum (Figure 2B).

#### The microbiome of the insulin-treated diabetic ileum

Insulin treatment resulted in a major reorganization of the microbial community of the ileum (Figure 2C). The ratio of *Firmicutes* did not change (48%), but the *Bacteroidetes* (scarce in the controls) made up 16% of the reads, accounting almost exclusively for the members of the genus *Bacteroides* (data not shown). The *Actinobacteria* were represented twice as strongly as in the diabetic samples (the order *Bifidobacteriales* 9% replacing the order *Corynebacterium* in the diabetic ileum). At the level of lower taxonomic units, characteristic signs of increasing microbial diversity and rearrangement of the microbiota were evident. Within the *Firmicutes*, the order *Clostridiales* (hardly detectable in the controls) was enriched to an astonishing 30% relative abundance. This order consisted essentially of two genera in equal representation, *Clostridium* and *Peptostreptococcus*. Insulin treatment was apparently deleterious for the order *Lactobacillales* in the class *Bacilli*: their number decreased 4-fold relative to the control and the diabetic ileum. The *Proteobacteria*, including the genus *Klebsiella*, the indicative signature of diabetes, were practically eradicated by insulin from the ileum of the diabetic rats. Only the order *Burkholderiales* represented the *Proteobacteria*, in 2% abundance. A notably high proportion of the microbial community (20%) remained unassigned in these samples. Although rationalization of the considerable changes in the various taxa as a result of insulin medication requires further study, it is clear that the microbial community of the ileum was rearranged and diversified substantially.

### The microbiome of the colon

#### The microbiome of the control colon

The number of *Archaea* (0.1%) found in the colon was still small relative to the domain *Bacteria*, but was 10-fold higher relative to that in the ileum. The phyla *Firmicutes* and *Bacteroidetes* predominated in the colon of the control rats (Figure 3A). These two phyla each comprised 40% of all the identified sequences, *Proteobacteria* (4% representation) and *Spirochaetes* (2%) were also present. The other microbial phyla displayed significantly lower relative abundances. At the genus level, the order *Clostridiales* consisted primarily of the genera *Clostridium* (11%), *Eubacteria* (6%), *Ruminococcus* (5%) and *Lactobacillus* (4%). The most abundant bacteria in the order *Bacteroidales* were categorized in the genera *Bacteroides* (18%), *Prevotella* (12%) and *Alistipes* (5%).

#### The microbiome of the diabetic colon

The abundances of the major phyla did not differ to a great extent in the colon samples of the diabetic and the control rats. The phyla *Bacteroidetes* (38%) and *Firmicutes* (37%) accounted for the overwhelming majority, followed by the *Proteobacteria* (11%). The *Bacteroides* (19%), *Prevotella* (15%) and *Alistipes* (4%) (not shown) were the major genera within the *Bacteroidetes*. The *Firmicutes* comprised mostly members of the genera *Clostridium* (12%), *Eubacterium* (6%), *Lactobacillus* (5%) and *Ruminococcus* (4%) (not shown). Major differences between the colon samples of the control and the diabetic rats were therefore hardly detectable at a genus level in the *Bacteroidetes* and *Firmicutes*. In contrast, the composition of the *Proteobacteria* differed significantly. Whereas members of the genera *Helicobacter*, *Wolinella* and *Desulfovibrio* were identified in the colon samples of the control rats, all of them in <0.5% abundance, their major representatives in the colon of the diabetic rats belonged in the genus *Klebsiella*. The relative abundance of 6% was significantly less than that observed in the diabetic ileum, but still noteworthy as compared with the control or insulin-treated colon communities (Figure 3B).

#### The microbiome of the insulin-treated colon

Insulin treatment of the diabetic rats resulted in significant differences in the colon microbiome, which rather resembled that in the ileum, although the differences relative to the controls were less pronounced (Figures 2C, 3A and 3C). The most obvious of the insulin effects on the microbiota was a general escalation of the diversity of the microbial community. The phyla *Bacteroidetes* and *Firmicutes* still ruled over the microbial landscape, totalling 44% and 30% of the population, respectively. Among the other phyla, the *Actinobacteria* were appreciably represented (8%), followed by the *Proteobacteria* (6%) and *Spirochaetes* (2%). It should be noted that the insulin treatment was accompanied by fundamental changes within the phylum *Proteobacteria*: their overall abundance decreased spectacularly, and the genus *Klebsiella* was basically eradicated, being replaced by the genera *Desulfovibrio* and *Bifidobacterium*, with a combined abundance of 4% (not shown).

## Discussion

The overwhelming majority of studies published to date on the role of enteric pathogens in the development of autoimmune diabetes [30]–[32] were carried out on faecal samples. Community-level investigations were recently extended to metagenomic [33], meta-proteomic [34] and metabolomics [35] descriptions of pathological alterations in the GI microbiome, but all involved faecal samples. Thus, these data do not necessarily reflect the microbiological events that take place in the various segments of the GI system under the pathological conditions leading to T1D and related enteric neuropathies. In order to explore the possible correlation between the diabetes-related gut region-dependent nitrergic myenteric neuropathy and the altered mesenteric capillaries [10]–[11] and the spatially-restricted distribution of the gut microbiota, we therefore carried out a metagenomic analysis of the luminal contents of the duodenum, ileum and colon of rats with STZ-induced diabetes and insulin-treated diabetes in comparison with control rats.

The proximal part of the GI tract does not harbour a rich microbial community: around 10^4^–10^5^ cells/g are found there [36]. The predominance of *Lactobacillus* strains, which characteristically inhabit the upper gut, [37] was not markedly different in the duodenal luminal contents of the diabetic and the insulin-treated diabetic rats. Accordingly, the composition of the duodenal microbiota did not indicate the development of a pathological enteric microenvironment. This is in good agreement with our earlier findings that in STZ-induced diabetes the duodenum was the only gut segment in which a decrease in the number of nitrergic myenteric neurones was not accompanied by a decrease in the total number of neurones,^10^ and the limited diabetes-related structural alterations in the mesenteric capillaries were completely prevented by insulin treatment [11].

In the ileum, where a significant decrease in the total number of myenteric neurones is accompanied by severe structural damage of the mesenteric capillaries in rats with STZ-induced diabetes, [10]–[11] the diabetes is characterized by a massive *Klebsiella* invasion. The *Klebsiella* are among the most common Gram-negative bacteria that cause severe intestinal inflammation in humans [38]–[39]. The mucosal inflammation results in a leaky epithelium; this allows the easier passage of bacteria through the intestinal epithelium, initiating a pathological cascade disturbing the intestinal immunology, a critical element in the development of the autoimmune T1D [40]–[42]. The aberrant microbiota that develops in the ileum in STZ-induced diabetes and the characteristic upsurge of the Gram-negative *Klebsiella* could therefore be directly associated with the inflammation and the development of the pathological microenvironment, leading to both autoimmune diabetes and diabetes-related enteric neuropathy. It is not clear whether the *Klebsiella* contribute directly to the advance of the disease, or whether the weakened intestinal barrier facilitates the growth of the *Klebsiella*, i.e. whether the *Klebsiella* are the cause or the consequence of the autoimmune disease. It is noteworthy that a close relative from the same family, the pathogenic *Escherichia coli*, has been identified as the main culprit in various GI tract diseases, including autoimmune diabetes, in humans [43]–[48]. We found that insulin treatment brought about a drastic reorganization of the microbial community in the luminal content of the ileum. The microbial diversity increased enormously. The *Protobacteria* were practically eliminated and the genus *Klebsiella*, the indicative signature of diabetes in the ileum, diminished. The *Proteobacteria* were represented only by the genus *Bordatella* (order *Burkholderiales*). These changes in the composition of microbiota may correlate with the glycaemic control and the restoration of the enteric microenvironment, which prevented the neuronal cell loss and the structural damage of the capillary wall. Insulin treatment rearranged the microbiota of the ileum so that it became very distinct from both the control and the diabetes populations. The microbiome of the ileum in insulin-treated diabetes resembled the colon community and insulin could not restore the control microbiota in either GI segment.

The relative abundance of the major phyla *Bacteroidetes* and *Firmicutes*, which predominate in both the human and the murine colon [33], [49] did not indicate a striking difference between the healthy and the diseased state or that in response to insulin replacement in comparison with the ileum. In contrast, the alterations in both the relative abundance and the composition of the phylum *Proteobacteria* here were pronounced. The insulin treatment of the diabetic rats brought about significant alterations in the colon microbiome, though the changes were less marked relative to the control and the diabetic rats. The most noticeable of these modifications was a general escalation of the diversity of the microbial community in both the ileum and the colon. A proliferation of microbial diversity is a general sign of a healthier environment in the human gut [22], [33]. Insulin replacement was accompanied by a fundamental change within the phylum *Proteobacteria* in the ileum and the colon. Their overall abundance decreased spectacularly and the genus *Klebsiella* was eradicated. Roesch et al. [49] reported that strains belonging in the genera *Lactobacillus* and *Bifidobacterium* were more abundant in genetically diabetes-resistant rats, which correlated well with the appearance of the order *Bifidobacteriales* in insulin-treated animals in our case. Their abundance was 9% and 7% in the insulin-treated ileum and colon, respectively. However, in mice a pro-diabetogenic, gluten-containing diet increased the relative numbers of the *Bifidobacterium*, the *Barnesiella* and the *Tanerella* [32].

Insulin appropriately controlled the hyperglycaemia in the colon and prevented the myenteric neuronal loss. Unlike the ileum, the structural alterations of the microvessels remained unchanged relative to the diabetic counterparts [11]. This suggests that, although hyperglycaemia can be controlled by appropriate insulin medication, some of the associated system irregularities persist. The fact that insulin did not restore normal conditions in the capillary endothelium or in the microbiota of the ileum or the colon further indicated that the main location of hyperglycaemia-dependent events is in the ileum, and studies involving only the colon or the stools are unlikely to give an overall picture.

## Conclusions

The regionally distinct alterations in the microbiome along the GI tract of rats with STZ-induced diabetes or insulin-treated diabetes correlated well with the regional manifestations of the diabetes-related enteric neuropathy and mesenteric capillary damage. This suggests that the myenteric neurones in a distinct gut segment are not simply targets of T1D, but rather active participants in the pathogenic pathways initiated by the regionally altered microbiota, a common environmental trigger for autoimmune diabetes and enteric neuropathy. The characteristic upsurge of the Gram-negative *Klebsiella* in the ileum could be directly associated with the inflammation, which is a decisive element in the onset of autoimmune diabetes. At the same time, the *Klebsiella* invasion could be a critical environmental trigger, initiating the pathological cascade in the gut wall and resulting in compromised neuro-immune interactions, enteric neuropathy and harmful GI syndromes. As the *Proteobacteria* exhibited the most significant changes seen in diabetes, we suggest that, rather than the predominant *Firmicutes* and *Bacteroidetes*, the less abundant *Proteobacteria* should be at the focus of the diagnosis and the potential therapy of T1D. More work is needed to establish clearly whether the *Klebsiella* are the cause or a consequence of the autoimmune disease. The massive invasion of the *Klebsiella* in the ileum and the related myenteric neuropathy raise the possibility that the *Klebsiella* might be regarded as a biomarker of the disease. The microbial diversity in both the ileum and the colon increased enormously in response to insulin treatment. The multiplicity of the bacteria was less profound in the colon, which might explain why the diabetes-related structural alterations in the mesenteric capillaries here were only partially restored in the insulin-treated diabetic rats. Although hyperglycaemia can be controlled by appropriate insulin replacement, some of the associated irregularities persist in the different intestinal segments and influence the outcome of the therapies.
